# Self-devised assistive techniques by university students with learning disabilities

**DOI:** 10.4102/ajod.v12i0.1106

**Published:** 2023-01-27

**Authors:** Ndakaitei Manase

**Affiliations:** 1 College of Education, Institute for Open and Distance Learning, University of South Africa, Pretoria, South Africa

**Keywords:** assistive technology, low-resource settings, learning disabilities, agency, conversion factors, coping strategies

## Abstract

**Background:**

Access to assistive technology for disabilities is limited in low-resource settings. Therefore, existing research focuses on accessibility challenges. This article focuses on how students with learning disabilities manage learning in the absence of assistive technology, a subject that receives less scholarly attention.

**Objectives:**

This article aims to provide insights on how students with learning disabilities manage learning in the face of limited access to assistive technology. It explores conversion factors that influence access to assistive technology.

**Method:**

This qualitative study used semistructured interviews to collect data from students with learning disabilities and respective university staff members who were recruited using convenience and snowballing techniques. Data were analysed thematically and supported by thick descriptions of experiences.

**Results:**

This study established that students have limited access to assistive technology, and they manage learning through self-devised means that are more socially than technologically or scientifically inspired such as self-affirmation, animal therapy, family support and prayer. Conversion factors, which affect ability by either enabling or constraining access to assistive technology, were identified at personal and institutional or environmental levels.

**Conclusion:**

The article concludes that even though students with learning disabilities devise unconventional assistive ways to manage learning, failure to access assistive technology is a capability deprivation that promotes inequalities.

**Contribution:**

This article provides insights that shift perspectives that students with disabilities are passive recipients of support; rather, they can be active agents who innovate nontechnological ways to manage learning in the absence of assistive technology.

## Introduction

People with disabilities often require augmentation to their functioning, considering that disability results from limited interaction between individuals with a health condition with personal and environmental factors (World Health Organization 2022). The International Classification of Functioning (ICF) framework, from which this understanding of disability is derived, notes that the body can be limited in performing meaningful activities because of an impairment and the demands of the external environment. Assistive technology systems, which the World Health Organization’s Global Cooperation on Assistive Technology defines as ‘the development and application of organised knowledge, skills, procedures, and policies relevant to the provision, use, and assessment of assistive products’ (Khasnabis, Mirza & MacLachlan 2015) can improve the participation of people with disabilities. Assistive products are:

[*A*]ny product (including devices, equipment, instruments, and software), either specially designed and produced or generally available, whose primary purpose is to maintain or improve an individual’s functioning and independence and thereby promote their wellbeing. (p. 2229)

Such understandings of disability and assistive technology are both medically and socially framed to stress the role of the body and the external environment in limiting participation. As such, this conceptualisation stresses the fact that, in securing assistive technology, focus should not only be given to what the body cannot do or struggles to do but also how the external environment influences what a person can do.

This article adopts the term assistive technology to refer to both assistive products and their application following the (2018) South African strategic framework on disability for post-school education and training that considers assistive technology ‘an umbrella term that includes assistive, adaptive and rehabilitative devices and services for persons with disabilities, which enable persons with disabilities and learning differences to attain independence’ (Department of Higher Education and Training [DHET] 2018:vii). This article further recognises the purpose of assistive technology as that of improving performance, productivity and independence while promoting students’ academic and general well-being. Accordingly, assistive technology comprises external products that students with disabilities require to minimise the challenges they face in meeting their cognitive, perceptive, social and physical needs for them to fully display their potential and be successful in their academic endeavours. Most importantly, assistive technology is useful in augmenting instructional arrangements, student engagement and student–teacher interaction for students with learning disabilities who face inflexible curricula, pedagogy, assessment and outcomes and learn under strenuous physical and psychological environments. The basic understanding is that learning disabilities can limit students’ potential, and therefore, they need supportive means to enhance their abilities.

The aim of this article is to provide insights on how students with learning disabilities manage learning amidst challenges in accessing assistive technology. This study thus examines how students experience learning, explores students’ access to assistive technology and analyses students’ coping strategies. The research questions are theoretically framed within the capability approach as follows: ‘how do students with learning disabilities exercise agency to manage learning in the absence of assistive technology?’ and ‘what and how do conversion factors influence students with learning disabilities’ access and use of assistive technology?’

## Perspectives on access to and use of assistive technology for disabilities

Existing knowledge shows that not everyone who needs assistive technology can access it, highlighting discrepancies between need and provision, which MacLachlan et al. (2018) view as rooted in social, demographic and structural factors. The World Health Organization (2021:1) highlights that only 1 in 10 people in need of assistive technology has access to it, with access more limited in low-resource contexts. Eide and Øderud (2009:152) allude to this fact by noting that only 5% – 15% of people who require assistive products in low-resource settings have access to them. The challenges are heightened for students with disabilities because many universities struggle to provide suitable assistive technology (Ndlovu 2021:10). This article acknowledges accessibility challenges and broadens discussions to include the alternative means that students with learning disabilities adopt to manage learning without proper assistive technology. The main argument in this article is that in the absence of assistive technology, students exercise agency to achieve multidimensional success despite various constraining factors.

Access to assistive technology is more limited for those with invisible disabilities such as learning disabilities because they are given little attention in rehabilitation programmes. Assistive technology is historically associated with physical and sensory disabilities (Boot et al. 2018:901). High-end specialised assistive technology that is specifically designed for people with learning disabilities is not accessible to many who should benefit from them (Fichten et al. 2020:29). Various factors are attributed to the low use of assistive technology for disabilities. These include limited availability of specialised assistive technology, a lack of funding to purchase devices and suitable software (Ndlovu 2021:10), a lack of knowledge on suitable assistive technology (Coleman et al. 2015:655) and a lack of training on the use of assistive technology (Judge & Simms 2009:34). Relatedly, in Harniss, Samant Raja and Matter’s (2015) special issue that focuses on access to and service delivery of assistive technology in resource-limited contexts, authors corroborate the fact that there are challenges in the provision of assistive technology owing to limited funds, weak policies and legislation, poor distribution and shortage of relevant expertise. Hence, most challenges are systemic and beyond an individual’s control.

Most higher education institutions in South Africa provide limited assistive technology and other support services to students with disabilities (Vincent & Chiwandire 2019:1). The South African apartheid regime that ended in 1994 contributed to some of these challenges because it limited funding to institutions that are now referred to as historically black universities. The regime’s discriminatory policies restricted budgets and expenditure for institutions of learning on the basis of colour, resulting in the unequal distribution of educational facilities and learning resources (Motala 2006:85; Sayed & Kanjee 2013:7). These provisioning disparities extended to special schools for students with disabilities that catered for nonwhite children. The schools were under-resourced and offered inferior education (Department of Education [DoE] 2001:9). The provisioning of assistive technology was thus affected by racially based budgetary restrictions, leaving many students with disabilities with unmet academic needs.

In addressing these inequalities, funding arrangements improved post-1994 when the democratic government encouraged mainstreaming students with disabilities and introduced various bursary schemes for post-schooling, including the (1999) National Student Financial Aid Scheme (NSFAS) for students from poorer backgrounds. The bursary also assists students with disabilities to purchase assistive devices, tuition, accommodation and meet other needs (NSFAS 2019:1). However, the bursary is not utilised by all deserving students because the selection criteria are limiting. Students qualify for the NSFAS disability grant based on low household income, only first-time undergraduates are eligible, and the grant does not cater for students at private institutions (NSFAS 2019:1). The criteria exclude most students from middle and upper-class backgrounds, those who get diagnosed after joining the university or those who register a disability way into the course. Even though the government effected considerable developmental programmes, including student grants, most universities still have limited infrastructure and resources owing to the inequalities of the apartheid regime. Access to assistive technology is thus limited by systemic factors that are beyond an individual’s control.

Financial constraints at a household or individual level also contribute to barriers in using assistive technology. Ruswa and Gore (2021:6) established that most higher education students in South Africa experience different deprivations that range from a lack of basic needs to resources for learning. Various factors contribute to such deprivation including the fact that 76% of the South African population lives in poverty and the unemployment rate is currently pegged at 34.4% (Statistics South Africa [Stats. SA] 2021:7). The popular #FeesMustFall protests in South Africa (Moloi, Makgoba & Ogutu Miruka 2017:212), where students were fighting against rising tuition fees and low government funding, reflect students’ financial woes. High university drop-out rates in South Africa are noted among students from low-income households who fail to secure funding (Machika & Johnson 2015:168). Financial constraint is thus a major factor that interferes with students’ ability to acquire educational resources such as assistive technology that enhances success in higher education. Students from low-income households are therefore likely to be deprived of the digital capital that is instrumental in making them technologically apt.

Literature also shows that psychosocial factors play a role in the use of assistive technology. Writing in the context of physical disabilities, Hemmingsson, Lidstrom and Nygård (2009:468) noted that some assistive technology can be markers of deviance among students. Hemmingsson and colleagues established that some students with disabilities abandon assistive technology because it exposes them as different from others. Desmond et al. (2018:439) stress the need for culturally and context-sensitive assistive technology that can meet one’s goals while accounting for the social environment in which one operates. As such, the culture on supporting students with disabilities is worth considering when purchasing assistive technology. Its use should be understood by both students with and without disabilities so that support services for students with disabilities should not appear as unfair advantage (Mullins & Preyde 2013:155); neither should it expose students as different. Bad experiences from negative attitudes towards students’ use of assistive technology can affect the utilisation of assistive technology, especially where learning disabilities are misconceived as being intellectually inferior. Hence, inasmuch as assistive technology is functional, there are psychosocial factors that need to be accounted for to avoid the rejection of assistive technology.

## Why is assistive technology important for students with learning disabilities?

Assistive technology is important to circumvent challenges that interfere with students with learning disabilities’ satisfactory undertaking of academic tasks. The demands of higher education, including reading high volumes of learning materials, excessive writing, long and busy lectures, conceptualising, executing and writing projects and many more, require assistive technology for students to cope (Lyner-Cleophas 2019:2). Learning disabilities negatively affect how one processes, transmits, stores, receives and retrieves information, posing the risk of the underdevelopment of skills that are necessary to undertake and succeed in formal education. Dyslexia, which mainly causes difficulties in understanding text (Hulme & Snowling 2016:731) and attention deficit hyperactivity disorder (ADHD), which mainly affects executive functioning (Brown 2009:37), are represented the most among this study’s participants and therefore get more attention in this article. The symptoms of dyslexia and ADHD can be comorbid (Lonergan et al. 2019:725), and they include, among others, a slow reading and writing pace, low reading comprehension capacity because of poor word recognition and decoding, slow articulation of tasks, poor organisation skills, forgetfulness, attention difficulties, hyperactivity, impulsivity, anxiety, difficulty and poor language output that include stammering and mind blanking (Hulme & Snowling 2016:731; Khasawneh 2021:221; Lonergan et al. 2019). Challenges with executive functioning that most students with ADHD experience can affect memory, causing one to struggle to prioritise and complete tasks timeously (Weyandt & DuPaul 2008:314). It also causes emotional distress that creates difficulties in coping with day-to-day demands (Weyandt & DuPaul 2008:314). The underdeveloped academic skills and the associated challenges can negatively affect academic performance and contribute to emotional distress. Against this background, there is a need for students with learning disabilities to use assistive technology that eases the management of disability, studies and life in general.

This article maintains that assistive technology is good for augmenting students’ strengths and potentials that might be limited by a learning disability, as it offers alternative modes of performing tasks that tend to bridge the gap between ability and the demands of the environment. This stance is supported by Floyd and Judge (2012:49), who noted that assistive technology improves reading, comprehension and the retention of information that also contributes to increased academic task performance. Speech-to-text software is useful for students with a slow writing pace, as they can dictate rather than write down text. In a systematic review by Shadiev et al. (2014:75) on how speech-to-text recognition can enhance learning, it was established that this technology improves comprehension, completion of homework and exam preparations. The programme is equally helpful for those with dyslexia who experience difficulties in expressing their thoughts on paper (Khan 2020:3). Learning disabilities can affect the coordination between what one thinks and what they end up writing down (Hoover, Kubina & Mason 2012:33), such that one can have the correct answers in mind while the written script contains illogical responses. Speech recognition software is therefore useful because it captures students’ thoughts and allows them to produce the kind of work that portrays their capabilities.

In addition, speech synthesisers are useful for students with slow reading pace, perception challenges and poor comprehension that emanate from decoding challenges. The text-to-speech software can improve reading and comprehension. This technology enables students to get audible versions of on-screen text by matching written text with preprogrammed audio-recorded vocabulary. ClassMate Reader (HumanWare Group, Drummondville, Quebec, Canada) and Kurzweil 3000™ (Kurzweil Education, Dallas, Texas, United States) are examples of text reading programmes that enable students to listen to an audio version of text and thus circumvent specific phoneme–grapheme decoding deficits (Floyd & Judge 2012:50–51). Screen readers read out text on the screen and have visual tools that highlight each word as it is read so that a student can hear and see what is read. The software uses speech synthesisers that convert scanned written documents into audible text where the scanned document can be read back to the user to reduce reading, comprehension or even sight challenges (Southwell & Slater 2013:35). The voice component in this software is useful for those with poor word recognition to listen with a better grasp, as noted by Floyd and Judge (2012:49) that reading assistive technology improves comprehension and the retention of information that also contributes to increased academic task performance. Students can also benefit from the read-aloud function that is available on most documents that are saved in the Portable Document Format. Most university libraries now have audio-formatted electronic learning resources. However, some old documents are not compatible with digital formatting, making them inaccessible for learning (Fichten et al. 2020:32).

Furthermore, there is a need for technology that aids planning and organising, as learning disabilities can affect executive functioning. Appointments and notes can be computerised to remind students of important information such as names, dates and times for exams, appointments and assignment deadlines. Personal data managers and free-form databases can allow students to store and retrieve information with ease and thus compensate for organising challenges (Adebisi, Liman & Longpoe 2015:17). Students can also benefit from mind-mapping technology, particularly those who struggle with the planning of assignments. Inspiration (TechEdology Ltd, Pewsey, United Kingdom) is one concept-making software that provides students with a framework to brainstorm, organise ideas, generate themes and formulate a workable storyline or outline that is useful when writing an assignment (Forgrave 2002:124). Students randomly brainstorm ideas on their assignments and input them in this organisational software that automatically rearranges them to create a logical outline that is useful in improving the quality of written assignments (Forgrave 2002:124). These prewriting organisers serve as artificial and external working memory systems and are viewed as more effective than traditional ways (Shah & Naqeeb 2020:31) because they provide clues on how to organise information and improve the coherence of ideas when writing. Poor spelling skills can be alleviated by spell checkers that are available on most word processors, where incorrectly spelt words can be highlighted and options for correct spellings are offered (Adebisi et al. 2015:16). Word processors also have a proof-reading facility that corrects grammar or predicts words while someone is typing a sentence (Adebisi et al. 2015:16). This is helpful for students to produce presentable work and it eliminates dependence on scribes or human spell checkers. Students can thus take shorter time to complete tasks and with less effort than without assistive technology.

## Theoretical framework: Understanding assistive technology within the capability approach

Amartya Sen’s capability approach frames theoretical discussions in this article, using the concepts capability, conversion factors and agency. The capability approach is a normative framework for human development that stresses that people should have actual opportunities for them to function in ways that support the achievement of the things they have reason(s) to value, given their circumstances (Sen 2014:527). Capability is the central concept of the capability approach, and it refers to one’s freedom or a set of real opportunities to promote or achieve valued doings and beings (Alkire 2005:121). Assistive technology can be regarded as a capability that enhances students’ chances of succeeding in higher education by improving functioning. Failure to avail the necessary technology is an inequality that contributes to the marginalisation of students with disabilities. Assistive technology is recognised for its generative (capability input) and transformative (facilitates achievements) capacity (Haenssgen & Ariana 2018:99). Inasmuch as technology is capability- or freedom-enhancing, its instrumental value in improving functioning and enabling better performance is subject to different conversion factors such as computer literacy, the social norms, technological environment and infrastructure, which Ahmed (2012:161) observes to be limiting in many developing countries. In this article, focus is given to conversion factors that interfere with the use of assistive technology and the achievement of academic goals. Conversion factors stand between a resource, ability and achievement, meaning that they influence (positively or negatively) how students access and use assistive technology. Existing literature notes that the role of technology can be limited by personal factors, where people fear to adopt it (Ahmed 2012:161); by social factors where, for example, societal norms prohibit women to communicate with men on a mobile phone (Haenssgen & Ariana 2018:108) or where women cannot benefit fully from digitally-projected voice-based messages because they must leave the front seats to men (Oosterlaken, Grimshaw & Janssen 2021:118); or by environmental factors, where governments control how assistive technology programmes should be implemented (Oosterlaken et al. 2021:118). Therefore, conversion factors are represented where people have limitations in benefiting from a resource or opportunity.

The analysis of students’ experiences of assistive technology extends to their agentic role in managing learning. Sen (1999:19) views an agent as ‘someone who acts and brings about change, and whose achievements can be judged in terms of her [*sic*] own values and objectives’. An agent therefore takes a participative role and actively works towards achieving what is valued. Students’ agentic role is analysed based on what they do in pursuing valued academic goals considering barriers to accessing appropriate assistive technology.

The strength of the capability approach lies in how it propels social justice and accounts for the process leading to achievements (Grunfeld, Hak & Pin 2011:152). The capability approach stipulates that judgements on how well a person is doing cannot be solely based on availed resources or achievements but on the process leading to achievements, because there are conversion factors that interfere with the ability to achieve. Therefore, judgements on the well-being of students cannot be entirely based on the availability of the disability unit (DU) and the grades they achieve but also on the learning experience. This holistic approach to evaluating students’ experiences provides a broader informational base in the designing of educational and student support policies. However, the capability approach is just an evaluation approach that does not provide prescriptions of what to do but offers guidance by characterising capabilities and inequalities. The capability approach thus lacks operationalisation and is weak in prescribing ‘feasible procedures of application’ (Gasper 2017:244). It requires complementary theories to apply it in specific contexts. Therefore, the findings of this article should not be regarded as prescriptive but rather explanatory.

## Research methods and design

This study uses perspectives of 15 university students, eight lecturers and five staff members from the DU and the Centre for Teaching and Learning at a public university in South Africa. All the students were registered and were on the DU’s database as having learning disabilities and receiving disability support. The main aim of the study is to explore how students with learning disabilities manage learning. Therefore, students were asked to narrate their university experiences in relation to the challenges they face and opportunities that support learning. Students were asked questions on the nature of disability they have, the kind of support they receive from the university and how they manage learning. Follow-up questions examined if students use any assistive technology, with further probing leading to examining the coping strategies they adopt. The main question directed at lecturers sought to examine their pedagogical practices, asking if they consider learning disabilities in teaching and assessing students with learning disabilities. Follow-up questions sought to understand if and how the DU engages them to meet the teaching and learning needs of students with disabilities. Staff members from the DU were asked about the university’s policy position on teaching, learning and supporting students with disabilities (general and specific) – the services they offer regarding disabilities, the challenges or limitations they face, challenges that are reported by students and lecturers and the measures taken to address them. The main question asked to the members from the Centre for Teaching and Learning was focused on if and how they work with the DU to ensure that students with disabilities are not disadvantaged in their academic endeavours.

The study adopts a narrative inquiry research design, which is a form of qualitative inquiry that focuses on experiences of a specific phenomenon (Polkinghorne 1995:5) – university learning with a learning disability in this case. Participants were purposively selected using convenience and snowballing techniques since students with learning disabilities were hard to reach. The university’s DU facilitated access to participants by allowing the researcher to approach students as they leave the facility, as students occasionally visit the unit for different purposes (convenience sampling). Students were asked to approach other eligible participants and referred me to them once they agreed to participate in the study (snowball sampling). The participation criterion was stressed that eligible participants should be registered students at that particular university who had registered a learning disability. Participants agreed to participate in the study by signing a written informed consent form after all the details about the study were explained and clarified to them. Pseudonyms are used to identify students in all the publications that use data from these participants so that their actual identity remains hidden in respect of the confidentiality clause in this study’s information sheet and consent form. Lecturers were approached individually either via e-mail or in person. Their actual identities are also hidden as part of the confidentiality and anonymity considerations. Members of staff who offer student support were identified according to the positions they hold.

This qualitative study used audio-recorded face-to-face semistructured interviews as a tool to collect students’ narratives on how they experience university with a learning disability, lecturers’ perspectives on teaching students with learning disabilities and support staff’s insights on how the university caters for the needs of students with disabilities. Data were collected in 2019 with telephonic coronavirus disease 2019 (COVID-19)-related follow-up interviews in 2020. Interviews with all participants were held in English, and there were no communication barriers since the study is situated within the higher education context where English is the primary medium of instruction. All interviews were transcribed and analysed manually by the researcher. The tape recorder used to record the interviews was kept safe during the data collection and analysis to prevent unnecessary data exposure to unintended audiences. The audio files were deleted from the tape recorder after all the interviews were transcribed and e-mailed to me. Interview transcriptions were saved in a password-protected zip folder on my laptop. The researcher is the only individual with access to participants and interview details.

### Data analysis

Thematic data analysis was adopted to make sense of students’ experiences of accessing and using assistive technology. Inductive and deductive reasoning were applied to come up with themes and weave them with existing literature and theoretical concepts that frame the study. The analytic process involved reading the transcribed data, generating codes from the transcribed interviews, manually developing themes, interpreting themes within the existing knowledge and theoretical framework and presenting the findings descriptively. Meanings from the collected data were inductively derived by categorising excerpts of transcribed narratives that are associated with accessing and using assistive technology. Critical engagement with the data led to the generation of codes, where parts of the interviews were systematically colour-coded, matching sections with similar meanings. Data were then categorised according to technology accessibility, associated challenges and coping techniques. Further analysis involved matching which data fits or not under the capability approach concepts guiding the study, which are conversion factors and agency. From this deductive analytic process, personal, institutional and social conversion factors were identified. Data excerpts that represent students’ agency were also identified.

Even though students had subjective assistive technology experiences, there are themes that were represented enough to be considered main findings. For example, the fact that almost all the participants indicated that they do not make use of assistive technology specific to alleviating the challenges imposed by learning disabilities qualified as a main finding. However, unique individual cases helped to uncover the essence of experiencing university with a learning disability since the study adopts a narrative framework that does not prioritise finding commonalities or quantifying experiences but deep meanings of lived experiences (Thorne 2000:68). As such, most findings are not presented numerically but descriptively.

### Ethical considerations

Ethical clearance was obtained from the University of the Free State’s General and Human Research Ethics Committee (ref. no. UFS-HSD2019/0038/2903/2507).

## Findings and discussion

The study established that students’ access to and use of assistive technology is mainly hindered by financial constraints at the national, institutional and individual or household levels. It is also noted that some students do not seek assistive technology for learning disabilities because of the reasonable accommodations they receive during examinations. The findings expose students’ marginalisation through undiversified learning modes and poorly presented learning content that force students to adapt unconventional ways of managing learning such as self-affirmation, family support, animal therapy and prayer. These findings reflect that students are active agents in their studies, where agency is demonstrated through devising non- or low-tech ways of coping with learning in the absence of high-tech assistive technology. Accessibility challenges are analysed within the capability approach as conversion factors. The identified conversion factors are personal (socio-economic status) and environmental (funding, low awareness and lack of inclusive teaching skills).

### ‘Disabled’ access to assistive technology for university students with learning disabilities

Even though the PhD study (Manase 2020) from which this article is drawn did not directly focus on assistive technology, the researcher was interested in exploring how students cope with learning given the fact that they have learning disabilities. Further probing on this subject provided insights on whether or what students use as assistive technology. From students’ narrated accounts, and consistent with Fichten et al.’s (2020:29) findings, it was established that not many of the students use high-tech assistive technology, particularly that which is specifically designed to address the challenges posed by learning disabilities. Assistive technology can be considered a capability input with both instrumental and intrinsic value for its contribution towards students’ independence, improved academic performance and good progress and ultimately well-being. Therefore, limited or lack of access to the necessary assistive technology reflects a capability deprivation that constrains functionings or achievements and perpetuates inequalities in higher education. To note here is the fact that the university under study had not implemented its own disability policy at the point of conducting this study. The draft policy that was availed to the researcher had no definitions of assistive technology and disability. Rather, it defined and explained impairments within the medical model of disability. Such positions can contribute to the accessibility challenges faced by students with invisible disabilities (learning disabilities included), especially where impairment is strongly linked to loss of physical function.

Most telling from students’ accounts are remarks that they never considered sourcing assistive technology since they benefit from adjusted examination conditions at the university. Students in this study are separated from others to write tests and examinations at a smaller and noise-proof venue. Other adjusted exam conditions include extra time, scribes who read and write down students’ responses, spell checkers and individual cubicles for those who use scribes or those who experience severe symptoms of a disability. All students are from departments that require them to produce a hand-written exam script. They are not allowed to use any computer-based assistive technology during exams.

The interview with the head of the DU revealed that the university has limited financial resources, and there are disabilities that are not catered for fully because of inadequate assistive technology, as noted in this excerpt that:

‘We are committed to accommodating our students with disabilities, but we sometimes encounter financial limitations. Right now, we are planning to get reading pens for our students with reading challenges, but we do not have enough funds for that … All this need financial resources that we currently do not have.’ (Head of DU)

As the above quote suggests, lack of institutional funding can impede the provision of assistive technology to students with learning disabilities. This is consistent with Lyner-Cleopas (2019) and Vincent and Chiwandire’s (2019) assertions that there are funding challenges in South Africa and DUs struggle to meet students’ needs. A lack of a clear institutional policy position that delineates disability and assistive technology can be attributed to the university’s limited provision of assistive technology for other disabilities such as dyslexia that requires reading pens, as mentioned by the head of the DU.

Financial constraints were also cited by students as a hindrance to accessing assistive technology, as illustrated in this excerpt that represents most of the students’ position:

‘It would be nice if I had something to help me with reading. I tend to be very slow at it … but those things are very expensive and some of the licenses need to be renewed now and then.’ (Tess, 3rd-year female student)

The sentiments noted above support views that most assistive technology is expensive and out of reach to many people in developing countries, as suggested by Eide and Øderud (2009:152). An effective reading pen can cost around R10 000, and one needs nothing less than R1500 to get an ordinary one. Affordability is a factor in accessing assistive technology. Only one student got a tablet through NSFAS that he mainly uses to type notes because he does not write well. The rest of the students reported that they were not aware of the NSFAS disability grant that can assist with the purchase of assistive technology. This reveals a lack of awareness and information on disability support services that can promote access to assistive technology.

Although all the students have access to computers (personal or university’s), only one has access to reading software that is specifically designed to alleviate the challenges posed by dyslexia. The student reported that he is fortunate that his parents secured the assistive software to aid reading and comprehension while studying. The assistive reading software provides independence and it enhances the student’s reading and comprehension skills. The usefulness of the reading software is demonstrated in how the students do not depend on someone else to read for him as is the norm when writing examinations. The student, who was diagnosed with dyslexia while in primary school pointed out that his ‘privileged’ background enabled him to have such personal arrangements, spotlighting a correlation between access to assistive technology and socio-economic status.

Another student with misophonia (a sound disorder) who is affected by any form of sound, uses personally sourced sound-blocking earphones during lectures. The earphones help her to follow the projected slides attentively since she cannot hear the lecturer’s voice. The student revealed that she puts extra effort to try and understand what is being taught because many lecturers use slides that are difficult to follow. Other students complain of how they ‘take little’ from the lectures because of the poor instructional delivery. This reflects the marginalisation of students through a lack of suitable and effective inclusive assistive technology in the form of curriculum aids. The same concern was raised by students in their experiences of online and remote learning during the COVID-19 pandemic, where most of them were digitally excluded through inaccessible and unusable learning content and resources. Students complained of cluttered PowerPoint slides and limited presentation of learning materials. It is problematic where diversity is not considered in instructional design and learning environments. Accessible and useable learning content benefits many students with diverse needs and promotes the equalisation of opportunities for people with disabilities (United Nations 1994:1).

### Students’ coping strategies to manage learning

Student’s narratives reveal that they manage learning through self-initiated coping strategies such as self-affirmation, family support, animal therapy and prayer. Students admitted that having a learning disability and learning without supportive technologies is challenging, to the extent of affecting both their academic and psychological well-being. What frustrates students the most is that their abilities are not fully reflected in the results they get. One major concern is that even though the adjusted conditions enable them to write examinations well, they struggle to learn and study for exams without the much-needed technological support. The heightened risk of failing induces anxiety and emotional distress that affect students’ class participation and exam preparations. Therefore, most students adopt self-affirmation to overcome emotional challenges that emanate mainly from the difficulties encountered in trying to meet their valued goal of progressing well academically. One student with dyslexia practises self-talk and affirms that, ‘I am able, I am not a quitter and I have come this far because I can do this’. Another participant, who acknowledged that she ‘struggles to understand lectures because they are fast-paced’ such that she ‘cannot do [*academic*] tasks effectively and as fast as others’, tells herself that:

‘I am normal. I understand everything even if I can’t get it now … if someone can do tasks in 30 minutes, it’s still fine if I do it in 45 minutes as long as I get the job done.’ (Brenda, Honour’s female student)

Self-affirmation is a survival tactic people adopt to deal with threats (Sherman 2013:834). In this study, students practise self-affirmation to manage threats to academic success. Even though Brenda in the cited quote above tries to be positive, her narrative highlights the difficulties posed by an inherent health condition and exacerbated by teaching practices that are not inclusive. Most students struggle in conventional lectures that are often administered under tight timetables and delivered with no conscious consideration for learning disabilities, as illustrated in this interview excerpt from a lecturer:

‘I never intentionally consider any special needs when teaching. I don’t think I need to adapt to any need because I don’t know what need is there to accommodate.’ (Male, Lecturer 4)

The sentiments expressed by the lecturer in the quote above were common among the lecturers who reported that they are not made aware of any disabilities to consider when teaching. In addition, lecturers complained that they are not capacitated to teach in ways that consider learning disabilities. These findings reveal that students are taught and treated as a homogenous group, yet they are diverse. For example, most students with dyslexia decode and comprehend information slowly (Snowling, Hulme & Nation 2020:503), making it difficult to acquire discipline knowledge or contribute meaningfully to debates during lectures if instruction is not diversified. One student puts it clearly that ‘[*m*]ost of the time lecturers rush information through’, such that ‘I am [*physically*] with the lecturer, but I have lost him’. Nonetheless, practising self-talk or self-affirmation demonstrates agency that promotes emotional well-being to avoid giving up and dropping out of university.

Family social support is another form of managing learning that students adopt. Love, care, acceptance and understanding were reported by students as valued kinds of support from their families, because some of these students find it very challenging to manage a disability, their everyday life and studies independently. One student who admitted that ‘I struggle to learn and I wanted to quit university and pursue archery’ appreciates how his mother encourages him to get a degree while pursuing his passion. Family members also send reminders for exam dates and times, which students value as an important form of support in the absence of effective assistive technology to support planning and memory.

Animal therapy appeared as another form of support that students adopt to cope with the demands of university work and emotions. Students use their pets, particularly dogs, as an audience when practising oral activities. To these students, dogs are not judgemental, so they can stammer, mispronounce words or have mind blanking moments without being teased or developing feelings of incompetency, which students reported as common experiences when presenting in front of their peers. In addition, those who experience periodic emotional difficulties depend on the companion of their pets to de-stress, as illustrated below:

‘I always call my dog my therapist. He is my natural support system. A dog doesn’t judge you the way people do. If I had a bad day here at the university, I grab my poor Jack Russell, hold it by its stomach, put it on my bed, close the door, sit there and then I start telling him all the horrible stuff that happened to me. So, my dog will be sitting there, coming closer to me if it sees that I am upset, lies next to me or lies on top of me because he is a small dog. He’s just supportive. I feel better afterwards.’ (Cici, 3rd-year female student)

Emotional disorders are common among people with learning disabilities (Nelson & Liebel 2018:44), of which depression was a common condition that affects students in this study. Students’ interaction with pets acts as a useful support system, and it has been proved to be beneficial where relationships with peers are threatened by fear or experiences of social ridicule (Keefer, Landau & Sullivan 2014:524).

Several students who identify themselves as Christians reported that they pray for strength, contentment, security and victory to overcome the challenges they face in performing academic activities. One student reported that ‘[*b*]elieving that God can enable me to do anything, motivates me’. Praying before an exam was cited as the most common practice by these participants. They trust the spiritual power to enable them to tackle academic tasks, knowing that they have no assistive technology to rely on during the exams. Accordingly, students’ agentic role is demonstrated through these means of managing learning. In fact, except for one student who took six years to complete a four year degree, others’ academic progress is good, and many had successfully completed their studies at the point of writing this article. In summary, most students adopt nonconventional measures to manage learning in the face of limited access to high-tech assistive technology.

### Conversion factors that influence the use of enabling technologies

Conversion factors are analysed in terms of their effects on ability to access assistive technology – enabling or constraining. This article identified personal and environmental or institutional conversion factors that influence access to assistive technology, and all of them are constraining. Students’ socio-economic status, which creates individual financial constraints that contribute to the unaffordability of assistive technology, is a personal conversion factor that negatively influences access to suitable assistive technology. Another personal conversion factor is students’ reluctance to seek information on assistive technology for learning disabilities because of the concessions they receive to write examinations under adjusted conditions. The identified environmental conversion factors include a lack of disability funding that ranges from limited access to the NSFAS grant to institutional budgetary constraints that limit the purchase of assistive technology for disabilities. Institutional conversion factors also include low awareness on assistive technology for learning disabilities, pedagogical practices that encompass lack of inclusive and diversified presentation of learning content, curriculum aids or instructional support. Instructional aids are assistive to students who struggle to access learning material that is presented through conventional modes. Failure to access or use assistive technology stands as a conversion factor with a diminishing effect on learning. Learning environments that do not support meaningful learning disable students, as disability entails not only inherent health conditions but limiting environments too.

## Conclusion

Assistive technology designed specifically for learning disabilities, although valuable, is accessed by few students. The university contributes largely to students’ accessibility challenges because it fails to provide the necessary assistive technology. Failure to provide assistive technology is a capability deprivation that promotes inequalities that disadvantage students with disabilities. Students’ learning opportunities and academic performance can be negatively affected; so is their independence, as some rely on others to produce an answer script for marking. Poor access to technological assistive elements compels students to adopt more social forms of support, which reveal that students are not passive recipients of support services but can be active agents who innovate ways to successfully manage learning. Even though the use of social means to manage learning is commendable, it reflects marginalisation that, if not critically assessed, may ‘paint’ students with learning disabilities as intellectually inferior. Yet there is no strong basis to suggest that students with learning disabilities have low intellectual abilities. Rather, students perform well under supportive conditions (Sarid, Meltzer & Raveh 2020:6). Therefore, universities should adopt teaching and disability models that account for students’ health conditions and the environment they operate in when designing policies. Therefore, the study recommends sustainable financial resources at the national and institutional levels to offer students with disabilities appropriate assistive technology. The study further recommends information and awareness-raising campaigns on assistive technology, particularly for invisible disabilities. The university should increase efforts to capacitate lecturers with inclusive instructional design skills to accommodate diversity of all forms. Further research should evaluate the effectiveness and sustainability of the nonconventional assistive ways of coping with learning for university students with learning disabilities.
